# The Ipswich Conference

**Published:** 1912-06-22

**Authors:** 


### The Ipswich Conference.

Much interest has been evinced in local government circles in the Conference on Tuberculosis at Ipswich, which took place on Thursday and Friday last week, when special reference was made to municipal control under the Insurance Act.

Dr. John Robertson, Medical Officer of Health for Birmingham, before reading his paper on "The Municipal Treatment of Tuberculosis," congratulated the town on the erection of a sanatorium and the equipment of a tuberculosis dispensary. He dealt with the statistics of the disease in the United Kingdom at the present time, showing that during the past fifty years it had been reduced by 55 per cent. in England, 49 per cent. in Scotland, while in Ireland, where preventive measures had not been actively enforced, up to a year or two since there was an actual increase in the percentage. It was, he said, a public duty to prevent the tuberculous patient from becoming infectious by seeing that adequate inspection, treatment, and supervision were always available. A determined attack must be made upon unhealthy trades with unhealthy workshops and bad housing conditions. He regarded the Garden City movement as one of the best steps which had been taken.

Dr. Dixon, of the Municipal Sanatorium and Tuberculin Dispensary, Birmingham, read a paper on "Tuberculin Treatment," full of data for municipal medical officers.

Dr. A. K. Chalmers, Medical Officer of Health, Glasgow, dealt with "The Insurance Act and Consumption," and referred to the fact that the Act did not select consumption as the sole objective of the benefit, but included all the various types of disease, while the power to extend sanatorium benefit to dependents would be welcome because it might be possible to deal in the country homes or otherwise with children and adolescents who might develop phthisis in after years unless there was such a provision. He gathered from the interim report which had just been issued that there would be two independent authorities dealing with tuberculosis in the same area—*i.e.*, the officer dealing with those who came under the Act and the sanitary authority dealing with those who did not. He suggested several anomalies likely to arise as a result.

Dr. Freemantel, County Medical Officer of Health for Hertfordshire, called attention to the lack of accommodation in beds, and suggested the use as sanatoria of hospitals now lying idle which had been erected for fever cases by the various sanitary authorities.

Dr. Ernest Storke, Medical Officer of Health, Bury St. Edmunds, lamented the lack of information and the interchange of opinion between various local authorities with regard not only to migratory patients but also as to the removal of tuberculous cows, and spoke generally on the effect of factory conditions in town life on the one hand and tuberculous milk in rural life on the other hand.

Dr. Buckham, of St. Helens, expressed the opinion that the municipalities' hopes of financial support from the Act were likely to be disappointed, and described as a myth the supposed power of the Insurance Commissioners to extend the benefit of sanatorium treatment to insured persons as they had not the money. It would have to come out of the rates.

Dr. A. M. N. Pringle, Borough Medical Officer of Health, Ipswich, stated it was in his opinion vitally essential that the support of the general practitioner should be enlisted by local authorities or the Commissioners.

Professor Sims Woodhead, Professor of Pathology, Cambridge University, dealt with the question of tuberculosis in relation to the milk supply. The first thing the local authorities must do was to get rid of all cattle which had tuberculosis of the udder. In Copenhagen, where they tackled the milk question more thoroughly, the percentage of deaths from tuberculosis had fallen much more rapidly than it had under the preventive measures hitherto in vogue here.

### Graduated Labour.

Dr. Marcus Paterson, Director of the Welsh National Scheme for the Prevention of Consumption, whose name appeared on the agenda as the author of a paper on "The Influence of Graduated Labour in the Treatment of Consumption," touched on his subject, and then entered into generalities in a humorous vein, which formed a striking contrast to other papers. Then the delegates left the Town Hall to be present at the opening of the new sanatorium, which we describe in another column.

